# An Application of Cold Atmospheric Plasma to Enhance Physiological and Biochemical Traits of Basil

**DOI:** 10.3390/plants10102088

**Published:** 2021-10-01

**Authors:** Faezeh Mirazimi Abarghuei, Mohammad Etemadi, Asghar Ramezanian, Ali Esehaghbeygi, Javad Alizargar

**Affiliations:** 1 Department of Horticultural Science, School of Agriculture, Shiraz University, Shiraz 71441-65186, Iran; mirazimi_f@yahoo.com (F.M.A.); ramezanian@shirazu.ac.ir (A.R.); 2 Department of Biosystems Engineering, College of Agriculture, Isfahan University of Technology, Isfahan 84156-83111, Iran; Esehaghbeygi@cc.iut.ac.ir; 3 Research Center for Healthcare Industry Innovation, National Taipei University of Nursing and Health Sciences, Taipei 112, Taiwan

**Keywords:** atmospheric cold pressure plasma, dielectric barrier discharge, ion leakage microbial load

## Abstract

This study aimed to investigate the effects of dielectric barrier discharge cold atmospheric plasma on the performance of basil (*Ocimum basilicum* L. cv. Genovese Gigante). Evaluations were carried out on several physiological and biochemical traits, including ion leakage, water relative content, proline and protein accumulation, chlorophyll and carotenoid contents, and antioxidant activity. Before planting, basil seeds were treated by cold atmospheric plasma under voltages of 10, 15, and 20 kV for 10, 20, and 30 min. The ion leakage rate in plants was significantly affected by the interaction between plasma and radiation time. In most treatments, the application of plasma significantly reduced the ion leakage rate. The application of plasma (10 and 20 kV) for 10 min significantly increased the relative water content of basil leaves. The maximum amount of total chlorophyll and carotenoid content occurred after applying plasma for 20 min with 15 kV. Furthermore, 10 and 15 kV treatments of atmospheric cold plasma for 10 min caused a significant increase in antioxidant activity. The highest total flavonoids were obtained after applying 15 kV treatments for 20 min and 20 kV for 30 min, respectively. Cold atmospheric plasma significantly increased the activity of peroxidase as an antioxidant enzyme. Moreover, the minimum and maximum values of microbial load based on logarithm ten were reached after applying 10 kV for 30 min and in the control group, respectively. In general, the results showed that dielectric barrier discharge cold atmospheric plasma could significantly improve basil plants’ physiological and biochemical traits.

## 1. Introduction

Basil (*Ocimum basilicum*) comes from the genus *Ocimum* (family: Lamiaceae) [1]. Basil is recognized as an important medicinal plant [2]. The developmental features of this species have a significant impact on its production, marketability, and consumption. The ingredients available in this plant’s leaves have made it highly valuable for therapeutic and culinary industries [3]. Despite the increase in the application of chemical drugs, there is a growing awareness about the benefits of medicinal plants and the drugs derived from medicinal plants. In some countries, they are considered an integral part of effective treatment [4]. Some of these medicinal plants, like basil, have significant roles in our dietary regime as vegetables. The basil plant can produce secondary metabolites typical of most of the Lamiaceae family species, and it can be planted in almost all tropical, semitropical, Mediterranean, and temperate regions [5].

The viability of seeds is a critical agricultural prerequisite for effective crop cultivation. Because of its dormancy, *O. basilicum* seeds have a low germination rate, resulting in a poor plant population establishment in the field, potentially reducing herb and oil yields [6]. Therefore, seed germination improvement has a direct impact on crop production. Various physical and chemical treatments can improve seed germination in a variety of food crops. Some of these methods include magnetic treatment, sunlight, ultraviolet light, and hot water soaking in the physical treatment methods and chemicals, fungicides, and hormones under the chemical treatments’ methods [7].

One recent processing technology is applying cold plasma in the agricultural and culinary industry [8]. Physical and non-thermal technologies such as plasma have been designed to meet culinary products’ safety and durability by quantifying various effects on quality and dietary features [7,9]. Literature reviews showed that cold plasma could greatly increase crop yields and germination rates [7,10,11]. For example, Bermúdez-Aguirre et al. [12] evaluated bacteria’s senescence in fresh products like lettuce, carrot, and tomato using atmospheric pressure cold plasma in argon. The results indicated that the degree of the microbial load decreased significantly after the application of cold plasma. Another study was aimed at evaluating plasma power on deactivating microorganisms in apple juice. The results showed that the application of a combination of argon and oxygen for 8 min significantly reduced the logarithmic cycle. The findings suggested that the highest deactivation degree occurs with the highest dose of plasma and the highest oxygen rate [8]. So far, plants’ biochemical characteristics and exterior/appearance have not been considered in this regard. New research has described the role of cold plasma in reducing the activities of oxidant enzymes such as peroxidase and oxidase polyphenol in storage conditions. These studies’ results indicate a decrease in the browning of fruits and vegetables [13]. Research has shown that the activity of oxidase polyphenol decreases by 90% after 180 s of cold plasma treatment, but peroxidase is more resistant and returns to 85% of its primary amount. This return is explained by the change of secondary enzyme structures, a decrease in the alpha helix of proteins, and beta structures. Plasma can also change the three-dimensional structure of proteins in trypsin enzymes due to breaking peptide bonds [8]. In a study on black pepper and oregano [14], 5 min of cold plasma treatment caused a significant eradication of color in plants. They attributed this reduction of color to the destruction of some photosynthetic pigments like carotenoids. In a study conducted by Grzegorzewski et al. [15], the effects of cold plasma treatment were evaluated on lettuce, and the results showed an increase in biosynthesis in the flavonoids of a lettuce leaf due to plasma treatment. They indicated that argon ions could destroy a significant portion of epidermises in lettuce if combined with active oxygen such as radical hydroxyl and radical oxygen, leading to the release of flavonoids and other combinations from central vacuoles in cells.

For this reason, as a result of plasma treatment, the amounts of protocatechuic acid, letheolyn, and desomethyn increased in a lettuce leaf [15]. Tappi et al. [16] investigated plasma effects on apple slices using a dielectric barrier system with a 15 kV and radiation treatment voltage for 10, 20 and 30 min. Qualitative indices such as solid material rate of the solution, titratable acidity, flavor index, color, and physiological properties have been explored. The browning reaction of apple and the activity rate of polyphenol peroxidase enzyme was reduced by 65% and 42%, respectively, but other qualitative indices did not change. In a study by Almeida et al. [17], plasma treatments ultimately maintained the phenol content, antioxidant capacity, color, and acidity of orange juice specimens. Plasma treatment increased the illumination index of the experimented orange juice specimen as compared to the control specimen [17].

Seeds are the reproductive organs of plants that have totipotency and are necessary for plant survival, dispersal, and sustaining of progeny. As reported in several studies, plasma treatment of seeds has a long-term effect on the vegetative growth of plants [18], and the control of vegetative growth is crucial for plant development and survival [19]. Therefore, this study examined the physiological and biochemical properties of basil plants after the treatment of basil seeds with dielectric barrier discharge atmospheric cold pressure plasma.

## 2. Results and Discussions

### 2.1. Microbial Load

Microbial contamination is a main problem that decreases shelf life, deteriorates taste, and causes food poisoning. The analysis of variance on the effects of plasma on the basil plant’s overall microbial load showed that the interaction between radiation duration and voltage significantly impacted the overall microbial load. In this study, the plasma application caused a significant decrease in the microbial load of basil plants. The minimum (2.42 CFU) microbial load was achieved based on logarithm ten after applying 10 kV for 30 min. The maximum (3.86 CFU) microbial load was achieved in the control treatment. Non-thermal plasma effects were assessed against several microorganisms. Previous studies have indicated that atmospheric cold pressure plasma technology can be applied to reduce or remove microbial activities in farm products such as strawberries, melons, pears, spices, and nuts [20], and the efficacy depended on the treatment time or dose used [21]. Deng et al. [22] reported that reactive oxygen species were major factors for microbial inactivation.

### 2.2. Relative Water Content

**Figure 1:**
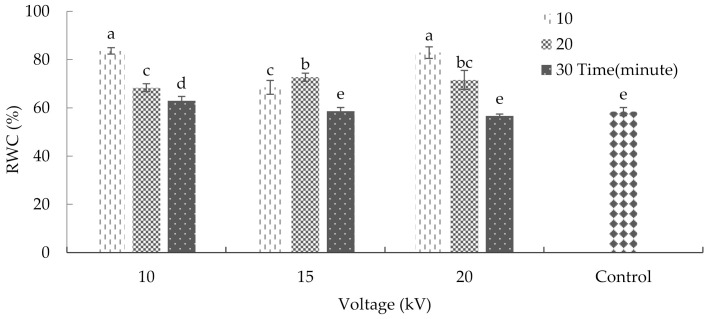
Plasma effects on the relative water content of basil. Error bars indicate standard error (*n* = 3). Different lowercase letters denote statistical differences between treatment groups at the 5% level according to Duncan’s test.

Leaf relative water content (RWC) is an essential indicator of plants water status; it displays the balance between the water supply to the leaf tissue and its transpiration rate [23]. This study showed that the combined application of radiation duration and voltage had significant effects on the RWC of the leaf (Supplementary Table S1). According to the results, the application of 10 and 20 kV voltages of plasma for 10 min significantly increased the RWC of the leaf (Figure 1). Previous results had shown that silicon improved the RWC in wheat under drought, which is similar to the effects of plasma radiation [24].

### 2.3. Ion Leakage

**Figure 2:**
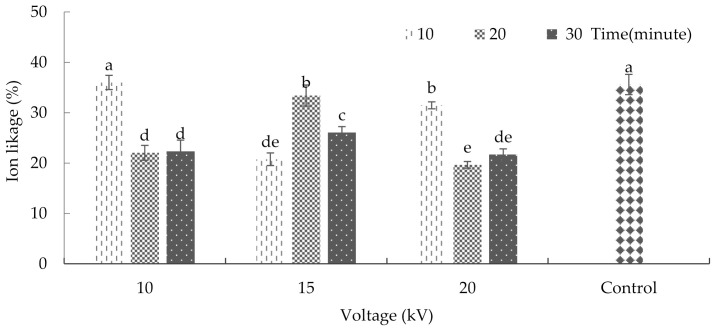
The effects of plasma on ion-leakage in basil. Error bars indicate standard error (*n* = 3). Different lowercase letters denote statistical differences between treatment groups at the 5% level according to Duncan’s test.

Cell membranes are one of the primary targets of many plant stresses. It is accepted that maintaining integrity and stability under stress conditions is an integral part of tolerance in plants. Furthermore, the rate of cell membrane injury induced by stress could be easily estimated by measurements of ion leakage from the cells [25]. The interaction between plasma and radiation significantly affected ion leakage in plants (*p* ≤ 0.01) (Supplementary Table S1). The comparison of mean values showed that plasma application significantly reduced ion leakage in plants in most treatments. The maximum rate of ion leakage averaged at 36% and 35.59% with respect to the treatment of seeds with 10kV plasma for 10 min and the non-treatment of seeds with plasma (control), respectively (Figure 2). Shi et al. [26] reported that ion leakage in bean leaves increased due to ultraviolet radiation, which causes stress to the plant. Using nitric oxide, they also found a possible means of reducing the damage incurred on the bean leaf’s tissue and membrane [27].

### 2.4. Total Protein and Proline

An accumulation of proline protects the cells against oxidative stress and upregulates the oxidative pentose phosphate pathway [28]. In addition, proline can also stabilize sub-cellular structures, quench active oxygen, and protect the cells against the disadvantageous effect of stress [29]. The results showed that plasma radiation did not cause a significant change in the total protein content of the samples compared to the control group (Supplementary Table S1). The analysis of variance regarding the effects of plasma on several biochemical properties of basil showed that the interaction between radiation time and voltage did not significantly affect the proline content (Supplementary Table S1). Hofmann et al. [27] found that ultraviolet radiation caused secondary oxidative stress (similar to plasma radiation) and increased the amount of proline in white clover flowers in a condition of full irrigation; however, it did not have any effect on proline content when low amounts of irrigation were administered and even caused a decrease in the proline of this flower [27]. Molinari et al. [30] investigated proline accumulation in sugarcane after exposure to drought. The results showed that the proline amount significantly increased after 6, 9, and 12 days [30].

### 2.5. Chlorophyll and Carotenoid

**Figure 3:**
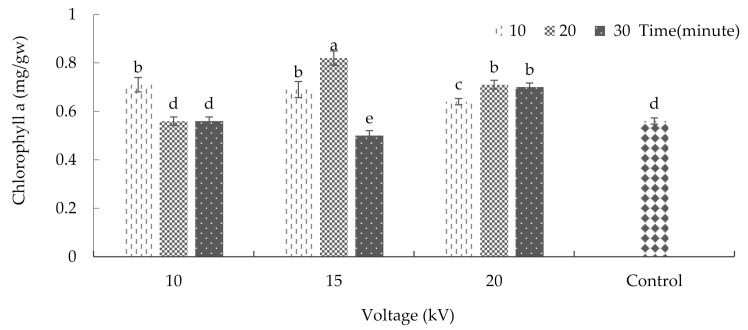
The effects of plasma on chlorophyll ‘a’ of basil. Error bars indicate standard error (*n* = 3). Different lowercase letters denote statistical differences between treatment groups at the 5% level according to Duncan’s test.

Chlorophylls, the main pigments in photosynthesis, are known to be linked with growth and productivity [31]. Moreover, changes in chlorophyll synthesis and biomass production are considered a secondary response to plant stress [32]. The analysis of variance revealed that the interaction between radiation time and voltage significantly impacted the amount of chlorophyll ‘a’ (*p* ≤ 0.01%) (Supplementary Table S2). This study showed that chlorophyll ‘a’ was affected significantly by plasma application (Figure 3). According to the results, after treating the seeds with 15 kV plasma for 20 min, the amount of chlorophyll ‘a’ reached its maximum, with an average of 0.82 milligrams per gram of fresh weight. However, as the results show, the chlorophyll ‘a’ content significantly decreased due to maintaining the 15 kV voltage and increasing the radiation exposure time from 20 to 30 min. Accordingly, the minimum amount of this pigment (with an average of 0.5 milligrams per gram of fresh weight) was observed after the application of a 15 kV voltage for 30 min.

**Figure 4:**
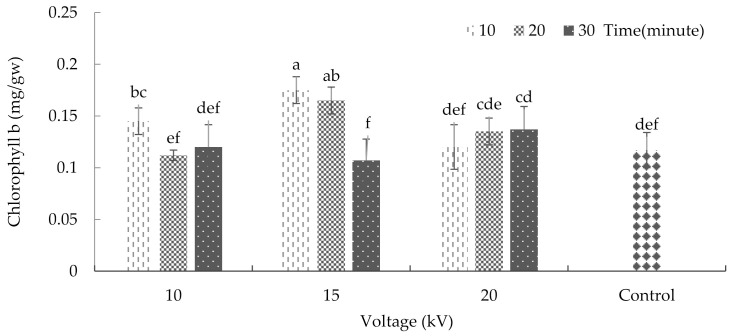
The effects of plasma on chlorophyll ‘b’ of basil. Error bars indicate standard error (*n* = 3). Different lowercase letters denote statistical differences between treatment groups at the 5% level according to Duncan’s test.

The interaction between plasma and radiation time significantly affected the amount of chlorophyll ‘b’ content (*p* ≤ 0.01%) (Supplementary Table S2). The current results indicate that the maximum amount of chlorophyll ‘b’ was achieved after applying 15 kV plasma for 10 and 20 min. These two treatments increased the amount of chlorophyll ‘b’ to 0.17 and 0.16 milligrams, respectively, per gram of fresh weight (Figure 4). Previous research has shown that ultraviolet radiation reduces the amount of chlorophyll ‘b’ more than chlorophyll ‘a’, and this shows a comparative susceptibility of chlorophyll ‘b’ in response to radiation [33].

**Figure 5:**
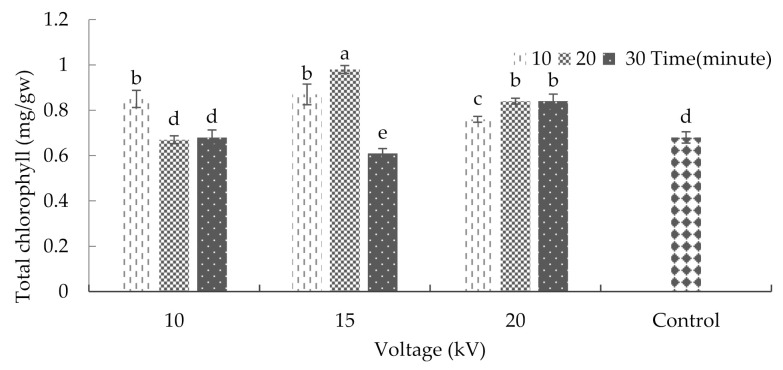
The effect of plasma on the total chlorophyll content of basil. Error bars indicate standard error (*n* = 3). Different lowercase letters denote statistical differences between treatment groups at the 5% level according to Duncan’s test.

The analysis of variance on the effects of plasma on some of the physiological characteristics of basil showed that the interaction between radiation time and voltage had a significant effect on the total amount of chlorophyll (Supplementary Table S2). The maximum and minimum amounts of total chlorophyll were 0.98 and 0.61 milligrams per gram of fresh weight, respectively, after the application of a 15 kV voltage for 20 and 30 min (Figure 5). In the case of harvested kiwifruits, a study showed that the chlorophyll content of individual fruits decreased during storage. However, the plasma treatment reduced the amount of damage done to the pigments. In part, this emanates from a minor deactivation of the chlorophyllase enzyme in the matrix [34]. Moreover, Sirgedaite et al. [35] have reported that seed treatment of Norway spruce with cold plasma caused a substantial increase in pigment.

**Figure 6:**
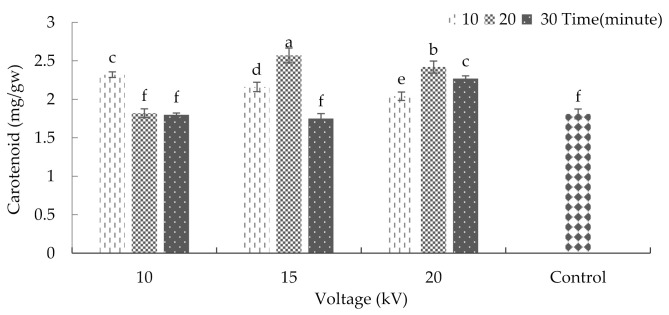
The effects of plasma on the carotenoid of basil. Error bars indicate standard error (*n* = 3). Different lowercase letters denote statistical differences between treatment groups at the 5% level according to Duncan’s test.

The interaction between radiation time and voltage was significant with respect to the carotenoid content (*p* ≤ 0.01%) (Supplementary Table S2). After treating the seeds with 15 kV for 20 min, the maximum amount of carotenoid was achieved (average = 2.57 milligrams per gram of fresh weight). Moreover, exposing the seeds to 10 and 15 kV for 30 min, 10 kV for 20 min, and to the conditions of the control group led to a significant decrease in carotenoid content (Figure 6). Previous research indicated that ultraviolet radiation could increase the carotenoid content in plants, which was in contrast to the decrease in chlorophyll content [33].

### 2.6. Antioxidant Activity

**Figure 7:**
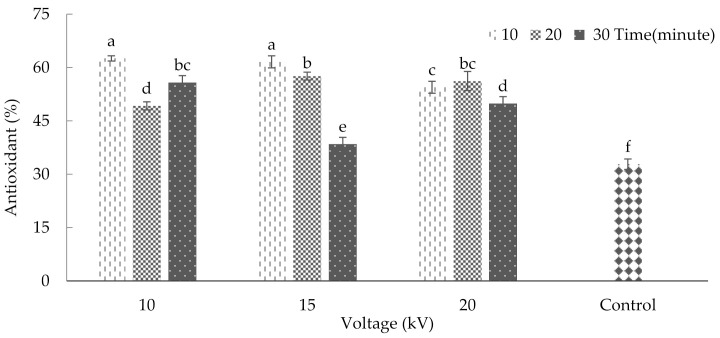
Plasma effect on antioxidant activity of basil. Error bars indicate standard error (*n* = 3). Different lowercase letters denote statistical differences between treatment groups at the 5% level according to Duncan’s test.

Plasma treatment had significant effects on antioxidant activity (*p* ≤ 0.01%) (Supplementary Table S3). The maximum levels of antioxidant activity (62.55 and 61.59) occurred after the application of 10 and 15 kV, respectively, for 10 min. These two maximum values were placed in one statistical category. The results also indicated a significant decrease in antioxidant activity in the control plants. The minimum antioxidant activity (average = 32.81) was observed in this group of plants (Figure 7). According to Won et al. [36], an increase in antioxidant activity in tangerine fruits occurred after applying cold plasma, which agrees with the current study. In another study, plasma contributed to increased antioxidant activity in dried walnuts [37]. Similar to our results, Muhammad et al. [38] reported that low N2 plasma exposure at the shorter time led to increased antioxidant activity, whereas the longer treatment time and greater flow rate led to a decline in the antioxidant activity. Generally, plasma-ROS should prompt the oxidation of the phenolic compounds responsible for the antioxidant activity. However, due to the opposite effect of tissue response mechanisms in kiwifruit, the ROS-induced oxidation was hindered [34].

### 2.7. Total Flavonoid Content

**Figure 8:**
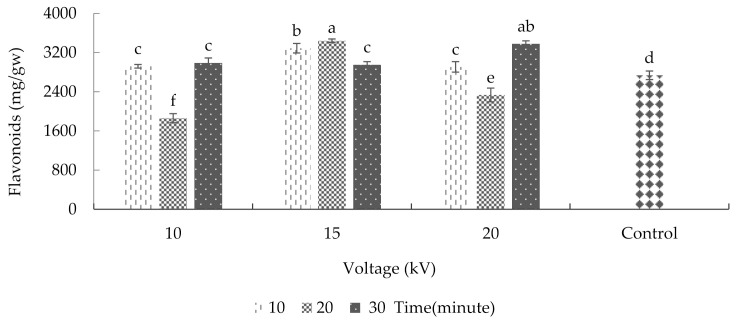
Plasma effect on the overall flavonoid of basil. Error bars indicate standard error (*n* = 3). Different lowercase letters denote statistical differences between treatment groups at the 5% level according to Duncan’s test.

According to the results, voltage and radiation time significantly affected the plants’ total flavonoid content (Supplementary Table S3). After treating the seeds with 15 kV for 20 min and 20 kV for 30 min, the maximum amount of total flavonoid content was achieved (3441.41 and 3378.68 micrograms per gram of fresh weight, respectively). Conversely, the minimum amount of total flavonoid (an average of 1859.76 micrograms per gram of fresh weight) was observed in 10 kV plasma treatment groups for 20 min (Figure 8). In research on lettuce, cold plasma treatment increased the leaves’ flavonoid content [15]. According to the same research, when argon ions are combined with ROS, such as with hydroxyl radicals or oxygen radicals, the epidermal cells of lettuce tissues can be damaged, leading to the release of flavonoids and other compounds from the central vacuole. For this reason, plasma treatment led to an increase in the amounts of protocatechuic acid, luteolin, and diosmetin in a lettuce leaf.

### 2.8. Sugar, Starch, and Total Phenol

The results showed that the plasma treatment did not significantly impact the amount of sugar, starch, and total phenol contents in basil plants (Supplementary Table S3). A previous study showed that plasma treatment on soya seeds at a radiation frequency of 13.56 MHz caused an increase of 16.51% in insoluble sugars than the control group [6]. In another research, oxygen plasma treatment reportedly changed the structural form of corn starch and potato (at molecular and super-molecular levels) [39]. Oxygen plasma on wheat starch can affect the carbonyl group by changing the crystal shape of hydroxyl groups [40]. Furthermore, low-pressure plasma has a relatively minor effect on the crystal structure of starch in rice [41]. Herceg et al. [42] reported that cold plasma application significantly increased phenol compounds in pomegranate. They demonstrated that when pomegranate juice receives cold plasma treatment, the active chemical species turn into charged particles and, thus, ultraviolet photons are created. These photons have sufficient electrical energy to break covalent links and stimulate several chemical reactions that may rupture the cellular membrane and facilitate hydrolysis or depolymerization of phenol compounds [42].

### 2.9. Super Oxidase Dismutase Enzyme, Catalase, and Peroxidize

**Figure 9:**
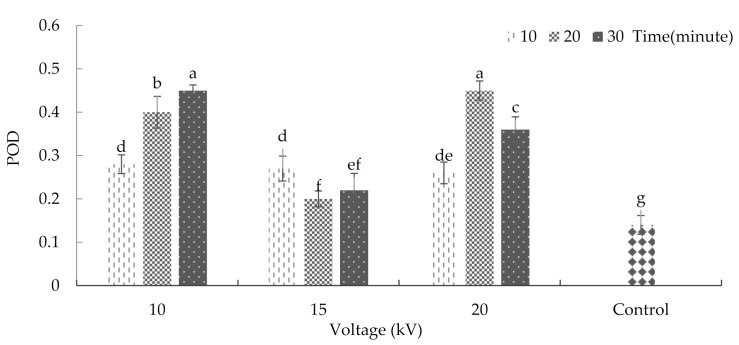
Plasma effect on peroxidase enzyme activity in basil. Error bars indicate standard error (*n* = 3). Different lowercase letters denote statistical differences between treatment groups at the 5% level according to Duncan’s test.

Cold atmospheric plasma (CAP), by producing reactive oxygen and nitrogen species (RONS), alters the redox homeostasis by controlling the activities of antioxidant enzymes such as peroxidase (POD), superoxide dismutase (SOD), and catalase (CAT) [43]. The effects of plasma on several biochemical properties of basil showed that the interaction between radiation time and voltage did not significantly affect the rates of super oxidase dismutase and catalase activities (Supplementary Table S4). However, the interaction between plasma treatment and radiation time significantly affected peroxidase activity (*p* ≤ 0.01%) (Supplementary Table S4). Figure 9 represents the results of plasma effects on peroxidase antioxidant enzyme activity. As the results show, the maximum peroxide antioxidant enzyme activity was achieved after 10 kV plasma treatment for 30 min or 20 kV plasma for 20 min. The minimum rate of this enzyme activity (with an average of 0.14 units per milligram of protein) was observed in the control treatment. The results indicated that cold plasma increases peroxidase antioxidant enzyme activity in basil. One of the reasons for the change in the enzyme activity after plasma application could be attributed to this treatment’s role in changing spiral areas of alpha and beta pages in proteins [44]. Meiqiang et al. [45] were among the first to investigate the effects of plasma on peroxidase activity. They used magnetic arc discharge plasma on tomatoes, and the results showed that plasma treatment could increase peroxidase enzyme activity. Adhikari et al. [43] also reported that the gene expression of SOD, POD, and catalase were dependent on the duration of treatment time with cold plasma.

## 3. Materials and Methods

### 3.1. Atmospheric Cold Plasma (ACP) System

**Figure 10:**
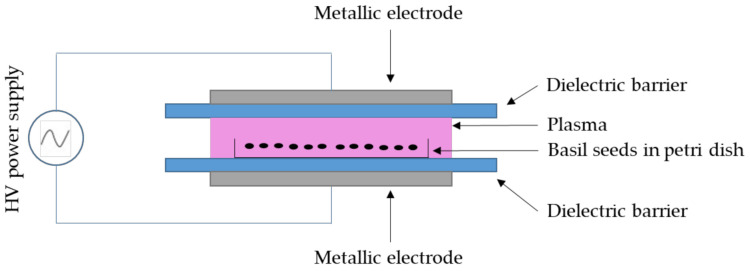
A schematic representation of the experimental set-up of the dielectric barrier discharges (DBD) plasma reactor for plasma treatment of basil seeds.

The Enhancedtech-18A plasma system developed by Kavosh Yaran Fan Pouya had a cylindrical chamber to create a DBD (Figure 10). The device had a power supply ranging from 1 to 20 kV, a frequency of 50 Hz and 20 kHz, a variable power of 0 to 50 kW, and a square pulse waveform. Two separate power supplies were used to improve performance. One power supply had a constant frequency of 50 Hz and a variable electric voltage of up to 20 kV, and the other power supply had a frequency of 20 kHz, a constant electric voltage of 20 kV, and a variable power of 0 to 50 kW. The simultaneous use of these two power supplies in this device facilitated the application of variable voltages, frequencies, and capacities. The dielectric barrier plasma in atmospheric pressure contained steel electrodes with intervals varying from 1 to 4 cm for higher conductivity. Plasma irradiation was set at 0, 10, 20, and 30 min, the electric voltage was 10, 15, and 20 kV, and a frequency of 50 kHz in 1 cm intervals.

### 3.2. Sampling, Culturing, and Storage Conditions after Plasma Radiation

Basil seeds (Genovese Gigante) were purchased from a reliable company. In this experiment, the seeds treated with atmospheric cold plasma were placed in Petri dishes (25 seeds to each petri dish) and were transferred to a growth chamber with a relative humidity of about 75%, night temperature of 19 °C, and day temperature of 25 °C. The seeds germinated and were placed in soil-less cultures depending on each treatment in separate pots. Following the growth of basil seedlings, the plants were harvested for analysis and were packaged in polypropylene plastic containers. In each package, 50 g of basil was placed. The specimens were kept in a refrigerator with a temperature of 10 °C and relative humidity of 85–90% for ten days. A specimen was taken out of the refrigerator daily to evaluate its properties [46].

### 3.3. Microbial Load

Following a method used by Valverde et al. [47], the experiment was performed with Plate Count Agar (PCA) to evaluate the microbial growth rate. First, 5.9 g of the culture medium was dissolved in 250 mL of distilled water. It was placed in a microwave to boil. In the next stage, the solution was autoclaved for 20 min at 121 °C. At this stage, 10 g of the herbal extract was placed in a plastic bag and, after adding 90 mL of saltwater, the plant tissue was smashed. At this stage, 1 mL of each specimen was added to 9 mL of 9% salt water before being shaken vigorously. One ml of the final solution was placed on a plate, and a layer of culture medium was added. After one minute, another layer of culture medium was added. The resultant mixture was first dried and then placed in an autoclave for three days at 30 °C. Finally, the Petri dishes were evaluated in terms of microbial populations, and the result was reported as the microbial load index [47].

### 3.4. Relative Water Content

To measure the relative water content of the leaf from each experimental unit, three leaves were selected from the middle part of each plant stem in the first step. Discs were sampled from these leaves and were weighed by a digital scale (with an accuracy rate of ±0.0001 g). The discs were then transferred to a Petri dish containing distilled water. The discs were maintained in a cool and dark place for 24 h. After extracting the leaves from distilled water, the extra water of the leaves dried, and the turgid weight of the leaves was measured. Finally, the relative water content of the leaf was calculated using Equation (1). In this equation, FW is the fresh weight of the leaf, measured immediately after sampling. DW is the dried weight of the leaf after being put in an oven. Finally, TW is the turgid weight after being placed in distilled water [48].

(1) $$\mathrm{RWC}\;(\%)=\frac{\mathrm{FW}-\mathrm{DW}}{\mathrm{TW}-\mathrm{DW}}\times 100$$

### 3.5. Ion Leakage

To measure the ion leakage, first, pieces of leaf with equal sizes were selected. Then, after adding 10 mL of distilled water and being placed on a shaker for 24 h, the EC1 was measured using an EC meter (Hanna Instruments model). In the next stage, specimens were autoclaved at 121 °C for 20 min. Then, after cooling down to 25 °C, EC2 was measured, and finally, ion leakage was calculated using the following Equation (2):

(2) $$\mathrm{EL}\;(\%)=\frac{\mathrm{EC}1}{\mathrm{EC}2}\times 100$$

### 3.6. Proline

In the first step, one gram of leaf specimens were placed in liquid nitrogen to grind them. The specimens were added to 5 milliliters of ethanol and were then centrifuged for 10 min with a speed of 3500 rounds per minute (rpm). The supernatant was transferred to a falcon. The proline concentration of the specimens was measured using the reagent solution ninhydrin (1.25 g of ninhydrin mixed with 30 mL of glacial acetic acid and 20 mL of phosphoric acid six molars). For this purpose, 1 mL of the extract was mixed with 9 mL distilled water and 5 mL ninhydrin reagent solution. Then, the specimens were placed in a hot water bath at 65 °C for 45 min. After the specimens cooled down, their absorption was read at a wavelength of 50 nm by the spectrophotometric method [49].

### 3.7. Total Protein

To prepare a protein extraction buffer, 500 mg of polyvinylpyrrolidone was added to 6.07 g Tris before being dissolved in 450 mL distilled water. In the next step, the acidity of this solution was lowered to 8 using 1-normal hydrochloric acid. Finally, distilled water was added to make the solution reach 500 mL. To extract protein from the specimens, 50 milligrams of the sample leaf were mixed in 2 milligrams of the buffer mentioned above at a ratio of 4:1. The resultant solution was centrifuged for 10 min at 4 °C. To measure the overall protein content in the specimens, the upper phase of the mixture was used according to the Bradford [50] method based on attachments being established between Coomassie Brilliant Blue G250 (in the reagent acid) with protein molecules. To prepare the reagent acid, 0.01 gr of Coomassie Brilliant Blue G250 was dissolved in 5 mL ethanol (96%) by a magnetic stirrer. Then, 10 mL of acid phosphoric (85%) was added to the solution, and, after stirring, distilled water was added to increase the final volume of the solution to 100 mL. The reaction mixture comprised 80 microliters extraction buffer, 20 microliters plant extract, and 5 mL Coomassie Brilliant Blue reagent. The optical absorption of each specimen was read after two minutes of stirring followed by 5 min of no motion at room temperature. The reading was done at a wavelength of 595 nm. In this experiment, the extraction buffer was used as a control group. Each specimen’s protein density was calculated and reported according to the optical absorption value and bovine serum albumin (BSA) standard curve.

### 3.8. Chlorophyll and Carotenoid of Leaf

The total chlorophyll content, type ‘a’ and ‘b’ chlorophylls, and carotenoids in the leaves were determined using the dimethyl sulfoxide method [51]. First, 0.1 g of fresh leaf pieces were placed inside an Erlenmeyer flask. Then, 7 mL of dimethyl sulfoxide was added, and the solution was placed in an incubator machine for 30 min at 65 °C. The clear extract and the leaf tissues inside the Erlenmeyer flask were discarded and, then, by adding dimethyl sulfoxide, the extract volume reached 10 milliliters. Finally, using the spectrophotometer method, the extract’s absorption was read at wavelengths of 645, 663, and 470.

DMSO was used as a blank. The specimens’ chlorophyll and carotenoid contents were calculated and reported as milligrams per fresh weight of leaf, using Equations (3)–(6), as Gross [52] presented. In these equations, $C_{a\;}\;$ stands for the amount of chlorophyll ‘a’, C_b_ stands for chlorophyll ‘b,’ and ‘a’ stands for the wavelength value of absorption (nanometer). V is the final volume of the consumed solution, and FW stands for the specimen’s fresh weight.

(3) $$\mathrm{Chlorophyll}\;a\;(\mathrm{mg}/g\cdot \mathrm{FW})=\frac{12.7\left(A_{663}\right)-2.69\left(A_{645}\right)\times V}{\mathrm{FW}\;}$$

(4) $$\mathrm{Chlorophyll}\;b\;(\mathrm{mg}/g\cdot \mathrm{FW})=\frac{22.9\left(A_{645}\right)-4.68\;\left(A_{663}\right)\times V}{\mathrm{FW}\;}$$

(5) $$\mathrm{Total}\;\mathrm{Chlorophyll}\;(\mathrm{mg}/g\cdot \mathrm{FW})=\frac{20.2\;\left(A_{645}\right)+8.02\;\left(A_{663}\right)\times V\;}{\mathrm{FW}\;}$$

(6) $$\mathrm{Carotenoid}\;(\mathrm{mg}/g\cdot \mathrm{FW})=\frac{1000\;\left(A_{470}\right)-1.82{\;C}_{a\;}-85.02{\;C}_{b}}{198}$$

### 3.9. Antioxidant Activity, Phenol, and Flavonoids

Methanol (70%) was used for extracting essential oil from the specimens. In all treatments, 1 g of powdered specimen was transferred to a falcon pipe. Then, 5 milliliters of solvent were added, and the specimens were preserved on a shaker at 200 rpm for 24 min. They were placed in a centrifuge operating at 6000 rpm for 15 min. In the next stage, the supernatant was collected and stored at −20 °C [53]. The samples were assessed for antioxidant activity using DPPH and spectrophotometric methods. In particular, the reduction in free radicals was a measure of antioxidant activity. To do this, 100 microliters of the prepared extract were added to 5 mL of the control solution (blank) at various densities. The solution was shaken vigorously for 10 s and then stored at room temperature in a dark environment. The absorption values of samples were read at 517 nanometers using a spectrophotometer (Epoch Microplate Spectrophotometer, BioTek Instrument, Winooski, VT, USA). Finally, the following Equation (7) was applied to calculate the antioxidant activity of the samples.

(7) $$\mathrm{Free}\;\mathrm{radicals}\;\mathrm{inhibition}\;(\%)=\frac{\mathrm{samples}\;\mathrm{absorption}\;\mathrm{value}-\;\mathrm{control}\;\mathrm{absorption}\;\mathrm{value}\;}{\mathrm{control}\;\mathrm{absorption}\;\mathrm{value}}\times 100$$

The total phenol content was measured by Folin–Ciocalteu reagent [54]. Accordingly, 100 microliters of each sample extract were mixed with 200 microliters of Folin (50%) and 2000 microliters of distilled water. After 3 min, 1000 microliters of sodium carbonate (20%) was added to the solution, shaken, and stored in a dark room. Then, the absorption of each sample was read using a spectrophotometer at a wavelength of 765 nanometers. In this experiment, the gallic acid standard curve was used for determining the total phenol content in the samples. Total flavonoid content was measured via colorimetry of chloride aluminum. For this purpose, 1 mL of each sample was selected, and 300 microliters of sodium nitrate was added along with 600 microliters of sodium chloride (10%). Six minutes after adding sodium chloride, 4 milliliters of NaOH (1 N) was added. This solution reached 10 mL by adding distilled water, and, finally, the optical absorption was read at 510 nanometers. The quercetin standard curve was used for determining the overall flavonoid content in the sample [55].

### 3.10. Soluble Sugars

To determine the amount of soluble sugars in the samples, 100 mL of each sample was ground to a powder with liquid nitrogen in a mortar. Then, 10 mL of ethanol (80%) was added before entering centrifuge tubes. The tubes were centrifuged for 10 min at 5000 rpm. Then, the supernatant was poured into an Erlenmeyer flask. Ten ml of ethanol were added. The solution was centrifuged again for 10 min at 5000 rpm, and the supernatant was added to the previously made solution. In the next stage, 25 microliters of 5% phenol solution and 125 microliters of dense sulphuric acid was added to the specimens. Finally, the light absorption rate was measured using the spectrophotometer method (model Epoch Microplate Spectrophotometer, BioTek Instrument, Winooski, VT, USA) in the wavelength of 490 nanometers. A glucose standard curve was used to determine the content rate of the soluble sugar in the specimens [56].

### 3.11. Starch

To measure the amount of starch in the samples, 200 microliters of distilled cold water were added to 260 microliters of perchloric acid (52%) combined with the residual sediments and the soluble sugars of the previous stage. After being shaken for 15 min, 400 milliliters of distilled water were added. The solution was centrifuged for 10 min at 5000 rpm. The supernatant was removed, and, again, 100 microliters of cold distilled water were added along with 130 microliters of perchloric acid (52%) to the remaining sediments of the solution. Then, the remaining sediments were centrifuged for 10 min at 5000 rpm. At this stage, the new supernatant was added to the previously formed solution. The solution was placed in ice for 30 min, and its volume was manually increased to reach 2 mL using distilled water. In the next stage, 200 microliters of Antron (2000 ppm) were added to 100 microliters of the above solution. The samples were placed in vials at 65 °C for 20 min after Antron was added. Using a spectrophotometer, the absorption of each sample was read at a wavelength of 630 nanometers [57].

### 3.12. Antioxidant Enzyme Activity

In order to prepare the required extract to assess the antioxidant enzyme activity, 0.5 g of fresh leaf tissue was homogenized in 2 mm of cold 50 mM potassium phosphate buffer (pH = 7), containing two mm ethylene diamine tetrastic acid (EDTA) and polyvinylpyrrolidone (PVP) (1%). The homogenous solution was centrifuged for 10 min at 4 °C and 10,000 rpm using a refrigerator centrifuge machine. Then, the resultant supernatant (the extract) was stored at −80 °C until further use. To measure the enzyme activity of superoxide dismutase, three milliliters of the reaction mixture containing 50 microliters of enzyme extract were used. The mixture also contained 50 mM potassium phosphate buffer (pH = 7.8), 13 mM L methionine, 75 mM nitro blue tetrazolium chloride (NBT), 0.1 mM EDTA, and four mM riboflavin. The riboflavin solution was separately made daily in a dark environment and added to the final stage’s reaction solution.

Sample cuvettes were shaken, and the mixtures were placed in a bright room (with four fluorescent 20-Watt lamps) for 15 min. Then, the reaction was stopped by powering off the lamps and placing the samples in the dark. A reaction mixture without an enzyme extract, which was not placed against the light, was used as a blank solution. Another reaction solution that was placed against light and produced the maximum color was used as the control solution. The absorption rate of each sample was read using the spectrophotometer at 560 nm. The superoxide dismutase enzyme activity was calculated and reported based on enzyme activity per gram of fresh weight. In order to measure catalase enzyme activity, the required mixture was prepared. The reaction mixture included 3 milliliters of a solution containing 50 microliters of extracted enzyme, 50 mM potassium buffer (pH = 7), and 10 mM hydrogen peroxide. The samples’ light absorption rate was read in 1 min with a time-frequency interval of 10 s and 240 nanometers. The catalase enzyme activity was calculated and reported based on an enzyme activity unit per one milligram of protein. The peroxide enzyme activity assessment was carried out using a spectrophotometer in 1 min, with a time-frequency interval of 10 s and 470 nm. The reaction solution contained 50 microliters of enzyme extract, 2.9 mL of potassium phosphate buffer (10 mM) (pH = 7), and 0.05 mL of 20 mM guaiacol. The reaction started after adding 20 microliters of hydrogen peroxide (40 mM). The peroxide enzyme activity was calculated and reported based on enzyme activity per one milligram of protein [58].

### 3.13. Leaf Color Analysis

Leaf color was evaluated by a colorimeter (model CR400/4P) by which the chroma index and hue angle were used for analysis. The chroma index indicates the saturation rate or color density. The hue angle is also an index of food color in which zero and 360-degree angles indicate red color, whereas 90, 180, and 270-degree angles indicate yellow, green, and blue colors, respectively. The chroma index and hue angle were calculated and reported according to the following Equations (8) and (9).

(8) $$\mathrm{Chroma}=\sqrt{a2+b2}$$

(9) $$\mathrm{Hue}=\mathrm{Arctan}\frac{b}{a}$$

### 3.14. Data Analysis

Data were analyzed by SAS software (Ver. 9.4). The analysis of variance was performed according to the completely randomized design for a factorial experiment that comprised two factors. Then, a *t*-test was carried out to compare the effects of the factors and the control. All experiments were repeated four times, with three plants in each replicate.

## 4. Conclusions

This study showed that the application of various voltages of cold plasma could significantly affect the microbial load, biochemical, and morpho-physiological indices of basil. The results also showed that the treatment of seeds by plasma at 10 kV for 30 min remarkably reduced the microbial load. Generally, in most of the measured properties, the treatment of seeds by plasma at 15 kV for 20 min increased the concentration of chlorophyll, carotenoid, and total flavonoid contents. The amounts of total phenol, proline, and protein in plants did not increase significantly. The application of 10 and 15 kV treatments for 10 min significantly increased the samples’ antioxidant activity. Some biochemical properties such as sugar content, starch, superoxidase dismutase antioxidant activity, and catalase were not affected significantly by cold plasma. Moreover, cold plasma application increased the relative water content of leaves but reduced the ion leakage in the cells. Altogether, the results showed that the changes in microbial load, biochemical, and physiological indices of basil were dependent on plasma generation voltage and plasma treatment time.

## Data Availability

The data presented in this study are available on request from the corresponding author. The data are not publicly available due to the project regulations for the investigators.

## Supplementary Materials

The following are available online at https://www.mdpi.com/article/10.3390/plants10102088/s1, Table S1: ANOVA table for effects of electric voltage and time on some of the physiological and biochemical indices of the basil, Table S2: ANOVA table for effects of electric voltage and time on some pigments of the basil, Table S3: ANOVA table for effects of electric voltage and time on some of the biochemical indices of the basil, Table S4: ANOVA table for effects of the effects of electric voltage and time on the antioxidant enzyme of the basil.
