# Supplementary material for: An Application of Cold Atmospheric Plasma to Enhance Physiological and Biochemical Traits of Basil
Source: Plants (Basel). 2021 Oct 1;10(10):2088. doi: 10.3390/plants10102088 (PMC8540659; doi:10.3390/plants10102088)
Supplement: Supplementary file 1 [file plants-10-02088-s001.zip › plants-1345806-supplementary.pdf]

Table S1. Summary of ANOVA table for effects of electric voltage and time on some of the physiological and biochemical indices of the basil.

Sources of variation	DF	MS			
		RWC	Ion leakage	Total protein	proline
Repetition	3	3.23	2.63	0.0001	2.82
Time	2	1091.2**	116.86**	0.011ns	58.75ns
Voltage	2	81.75**	25.04**	0.012ns	13.01ns
Time*Voltage	4	135.49**	229.32**	0.006ns	50.23ns
Error		4.94	1.99	0.006	18.16
CV%		3.19	5.44	7.51	13.43

*, ** and ns; denote the significance level in probability level 5%, 1%, and non-significance difference, respectively.

Table S2. Summary of ANOVA table for effects of electric voltage and time on some pigments of the basil.

Sources of variation	DF	MS			
		Chl a	Chl b	Total chlorophyll	Carotenoid
Repetition	3	0.00016	0.00032	0.00049	0.0015
Time	2	0.0392**	0.0019**	0.056**	0.351**
Voltage	2	0.019**	0.0018**	0.027**	0.21**
Time*Voltage	4	0.047**	0.0024**	0.069**	0.411**
Error		0.00056	0.00027	0.0008	0.0038
CV%		3.6	12.31	3.57	2.91

*, ** and ns; denote the significance level in probability level 5%, 1% and non-significance difference respectively.

Table S3. Summary of ANOVA table for effects of electric voltage and time on some of the biochemical indices of the basil.

Sources of variation	DF	MS				
		Antioxidant	Phenol	Flavonoids	sugar	Starch
Repetition	3	2.15	0.0000028	9067.01	4.18	1.47
Time	2	395.42**	0.0000041ns	1127793.9**	9.31ns	166.69ns
Voltage	2	34.54**	0.0000009ns	1226709.2**	0.42ns	126.07ns
Time*Voltage	4	216.7**	0.000012ns	909360.5**	3.28ns	43.38ns
Error		3.06	0.000009	7520.31	3.56	35.21
CV%		3.24	13.41	2.99	2.66	7.19

*, ** and ns; denote the significance level in probability level 5%, 1%, and non-significance difference, respectively.

Table S4. Summary of ANOVA table for effects of the effects of electric voltage and time on the antioxidant enzyme of the basil.

Sources of variation	DF	MS.		
		SOD	CAT	POD
Repetition	3	7	0.00089	0.0048
Time	2	32.48^{ns}	0.072^{ns}	0.023**
Voltage	2	33.29^{ns}	0.021^{ns}	0.073**
Time*Voltage	4	18.8^{ns}	0.085^{ns}	0.026**
Error		18.18	0.00078	0.00076
CV%		2.76	8.75	8.53

*, ** and ns; denote the significance level in probability level 5%, 1% and non-significance difference respectively.
